# Animal Models of Diabetes-Associated Renal Injury

**DOI:** 10.1155/2020/9416419

**Published:** 2020-05-20

**Authors:** Zahra Samadi Noshahr, Hossein Salmani, Abolfazl Khajavi Rad, Amirhossein Sahebkar

**Affiliations:** ^1^Department of Physiology, Faculty of Medicine, Mashhad University of Medical Sciences, Mashhad, Iran; ^2^Neurogenic Inflammation Research Center, Mashhad University of Medical Sciences, Mashhad, Iran; ^3^Halal Research Center of IRI, FDA, Tehran, Iran; ^4^Biotechnology Research Center, Pharmaceutical Technology Institute, Mashhad University of Medical Sciences, Mashhad, Iran; ^5^School of Pharmacy, Mashhad University of Medical Sciences, Mashhad, Iran

## Abstract

Diabetic nephropathy (DN) is the main factor leading to end-stage renal disease (ESRD) and subsequent morbidity and mortality. Importantly, the prevalence of DN is continuously increasing in developed countries. Many rodent models of type 1 and type 2 diabetes have been established to elucidate the pathogenesis of diabetes and examine novel therapies against DN. These models are developed by chemical, surgical, genetic, drug, and diet/nutrition interventions or combination of two or more methods. The main characteristics of DN including a decrease in renal function, albuminuria and mesangiolysis, mesangial expansion, and nodular glomerulosclerosis should be exhibited by an animal model of DN. However, a rodent model possessing all of the abovementioned features of human DN has not yet been developed. Furthermore, mice of different genetic backgrounds and strains show different levels of susceptibility to DN with respect to albuminuria and development of glomerular and tubulointerstitial lesions. Therefore, the type of diabetes, development of nephropathy, duration of the study, cost of maintaining and breeding, and animals' mortality rate are important factors that might be affected by the type of DN model. In this review, we discuss the pros and cons of different rodent models of diabetes that are being used to study DN.

## 1. Introduction

**Figure 1:**
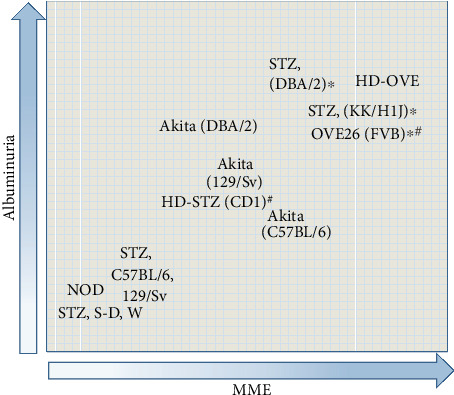
Albuminuria and renal histology in type 1 diabetic model. Notes: the level of albuminuria and mesangial matrix expansion is exhibited in each type 1 diabetic animal model. ∗Animals with nodular glomerular lesion. ^#^Animals with tubulointerstitial fibrosis. Abbreviations: S-D: Sprague Dawley.

**Figure 2:**
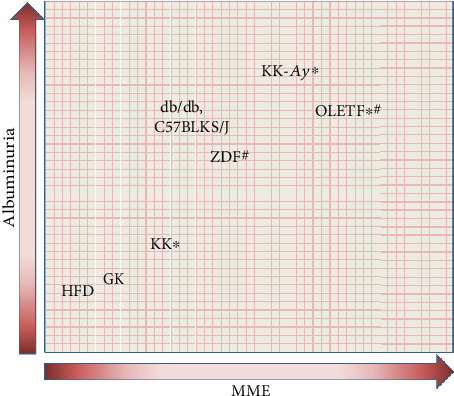
Albuminuria and renal histology in type 2 diabetic model. Notes: the levels of albuminuria and mesangial matrix expansion are exhibited in each type 1 diabetic animal model. ∗Animals with nodular glomerular lesion. ^#^Animals with tubulointerstitial fibrosis.

**Figure 3:**
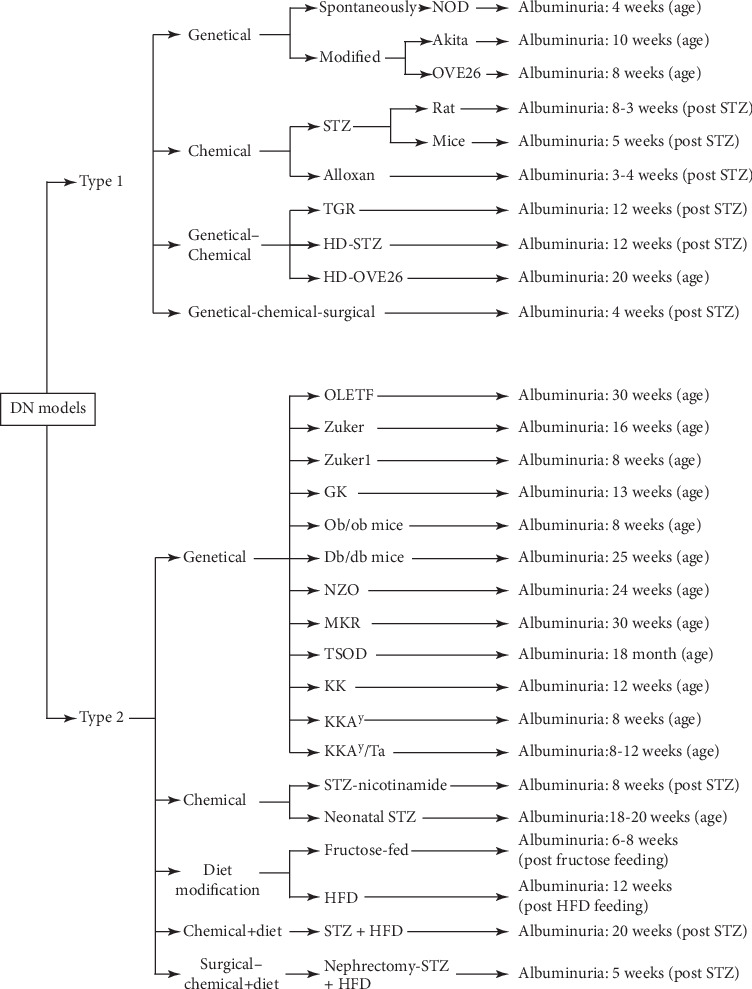
Albuminuria in DN models.

Diabetes is a progressive systemic disorder that may lead to diabetic nephropathy (DN) which is a major microvascular complication of the disease and the leading cause of end-stage renal disease (ESRD) [1]. To unravel the pathogenesis of DN and/or test novel therapies, several experimental animal models have been used [2]. These models are produced using chemical, surgical, and genetic (spontaneous development and manipulations) interventions; diet/nutrition alterations; or combinations of multiple methods [3, 4] (Tables 1 and 2). An ideal model of DN would display all DN features including reduced renal function (<50%) and albuminuria (>10-folds) as well as DN-induced histopathological changes such as mesangial expansion, mesangiolysis, and nodular glomerulosclerosis, observed in humans [5] (Figures 1 and 2). Since the incidence of diabetic nephropathy symptoms differ among experimental models, it is important to identify the appropriate model based on the type of study (Figure 3).

Large animal species, such as pigs and dogs, and humans share similar DN properties like decline in renal function and tubulointerstitial fibrosis [6]. Also, as such studies are prolonged experiments (e.g., >2 years in dogs) and impose greater husbandry costs, these models have few advantages over rodent models; thus, rodents are the preeminent species in preclinical research on nephropathy [7]. Mice are the most widely used species in preclinical research, as they rapidly breed and are relatively cheap to house; however, they tend to be resistant to the DN development [8]. Before the advent of genetic modifications in mice in the 1980s, rats were the most commonly used species in DN research [9]. Compared to mice, rats are greater in size and provide adequate renal tissue and blood for analysis. Furthermore, rats were proven to possess genes that are more relevant to human disease and they are more susceptible to many diseases including hypertension, cardiovascular diseases, and renal failure compared to mice [10–12].

### 1.1. Type 1 Models of Diabetic Nephropathy (T1DN)

Type I diabetes mellitus (T1DM), also referred as insulin-dependent diabetes or juvenile diabetes, is a chronic autoimmune disorder that is rooted in genetic susceptibility of individuals and is triggered by environmental factors. In the T1DM, the *β*-cells of the islets of Langerhans in the pancreas were attacked by the immune system, resulting in destructions or damages that are sufficient to reduce and eventually eliminate insulin production [13]. When more than 80% of *β*-cells are destroyed, as insulin secretion decreases, production of glucagon by the adjoining *α*-cells is increased. Insufficient levels of insulin along with excess glucagon production lead to hyperglycemia and ketoacidosis. Type 1 diabetes is characterized by activation of T lymphocytes and distinguished from type 2 (non-insulin-dependent diabetes) by the presence of autoantibodies, insulin dependence, genetic background, and insulitis.

### 1.2. Genetic Models of T1DN

Genetically modified animals facilitate mechanistic studies of pathological changes that cannot be evaluated in humans. In such animals, genes of the proteins that are thought to play key roles in glucose metabolism are manipulated. Knockout or transgenic animals express certain proteins at much lower or higher levels, respectively [14, 15]. In addition, knockout or overexpressed genes can help in explicit determination of the role of specific molecules and mechanisms in DN and allow investigation of novel therapeutic candidates for the treatment of DN. Genetic models include spontaneously developed or genetically modified animals [16].

### 1.3. Animal Models of Spontaneous DN

Animal models of spontaneous DN are established by breeding of the animals which develop DN due to genetic abnormalities. Spontaneous DN models are reliable because the renal abnormalities observed in these animals resemble the human DN characteristics. These models are not widely available, their feeding and breeding are rather difficult, and they require long modeling cycle and high cost. However, application of these models is becoming increasingly extensive [4]. Nonobese diabetic (NOD) mouse, biobreeding (BB) rat, and LEW.1AR1/Ztm-iddm rat are the most commonly used spontaneous models of type 1 diabetes in the studies [17, 18].

### 1.4. NOD Mouse

The NOD mouse develops spontaneous autoimmune diabetes with characteristics similar to those of human type 1 diabetes and is regarded as a hypoinsulinemic type 1 model of diabetes. Development of diabetes starts at the age of 3-4 weeks in NOD mice; most of females develop diabetes by week 40, but confirmed diabetes in males has dramatically lower incidence [19]. Application of NOD mice for DN studies was not as frequent as chemically induced animal models due to the rather long time required for the onset of diabetes. In addition to the development of only minimal renal abnormalities, spontaneous renal disease with uncertain etiology has been shown in NOD mice. In this model, lymphocytes infiltrate into the kidney but kidney tissue damage only manifests following diabetes development [20, 21]. Increased urinary excretion of albumin has been reported in these animals 4 weeks after diabetes onset [22].

### 1.5. BB Rat

BB rat is the spontaneously diabetic rat presenting insulin deficiency secondary to autoimmune *β*-cell destruction. Diabetes develops with equal severity and frequency among female and male. Diabetes occurs suddenly at about 8-16 weeks of age. Within a few days after diabetes onset, BB rats develop renal abnormalities including enhanced GFR and thickening of the glomerular basement membrane (GBM) without significant albuminuria or mesangial changes [23, 24].

### 1.6. Genetically Engineered Diabetic Mice

#### 1.6.1. Akita Mice

Akita mice are genetically engineered species susceptible to develop type 1 diabetes; this genetic engineering involves mutations in the insulin genes, which cause accumulation of misfolded insulin protein in pancreatic *β*-cell, leading to diminished insulin secretion capacity and type I diabetes [25, 26]. Glucose levels are significantly elevated in 4 week old of age, and albuminuria tended to increase at this time and are significantly increased after 10 weeks of age [27].

Genetic background is a key feature of DN development in Akita mice that influences the severity of renal injury. DN was observed following induction of Akita mutation in C57BL/6, DBA/2, and 129/SvEv strains; however, 129/SvEv and DBA/2 strains are more susceptible to nephropathy. Though these mice develop similar degrees of hyperglycemia, manifestations of nephropathy vary among them. For example, increased mesangial matrix was only observed in C57BL/6 and 129/SvEv mice. DBA/2 mice had higher levels of albuminuria compared with the others [28, 29]. Regardless of the strain, no studies demonstrated structural alterations similar to those observed in advanced human DN including mesangial matrix expansion, mesangiolysis, and nodular glomerulosclerosis. Importantly, female Akita mice develop mild diabetes compared to male ones. It was shown that the Akita mice develop more pronounced and prolonged hyperglycemia compared to mice with diabetes induced by STZ [29, 30].

#### 1.6.2. OVE26 Mice

OVE26 mice are transgenic animals that elicit calmodulin overexpression in pancreatic *β*-cells, insulin production deficiency, and type I diabetes. During the first week of age, the OVE26 mice develop diabetes and they can survive over one year with no insulin treatment. OVE26 mice exhibited significant albuminuria by 8 week of age [31]. Overexpression of calmodulin on the FVB background exhibits mesangial matrix expansion, global glomerulosclerosis, reduced podocyte number, renal fibrosis, and >10-fold increase in albuminuria by 6 months of age [32]. If the mutation of the calmodulin gene is induced in the DBA/2 or C57BL/6 strains, all overt features of DN will significantly diminish despite the susceptibility of DBA/2 mice towards DN development [31, 33]. Therefore, a significant limitation of these mouse models is the requirement of expression of such mutations in the FVB mouse strain to achieve the desired DN features.

### 1.7. Chemical Models of Type 1 Diabetic Nephropathy (T1DN)

Alloxan and streptozotocin (STZ) are frequently used for inducing T1DM and consequently, DN. These chemicals, which are both structurally similar to glucose, enter the pancreatic *β*-cells via the GLU2 transporter, but they are not recognized by other glucose transporters. This explains the relative toxicity of alloxan and STZ towards *β*-cells, since these cells express relatively high levels of GLUT2. The diabetogenic effects of alloxan and STZ are mediated by production of reactive oxygen species (ROS), finally resulting in necrosis of pancreatic *β*-cells. As time progresses, hyperglycemia culminates in diabetic renal damage [34].

### 1.8. STZ-Induced DN

STZ is an anticancer drug derived from *Streptomyces achromogenes* that is clinically used in the treatment of pancreatic *β*-cell carcinoma; also, STZ is widely used for induction of T1DM in rodents. STZ has toxic effects on *β*-cells as they have high levels of GLUT2 on the membrane but low capacity to scavenge free radicals and low NAD^+^/NAD ratio (6). In *β*-cells, STZ is broken down into methyl nitrosourea and glucose. Due to its alkylating properties, STZ modifies DNA fragments [35]. The kidney, liver, and intestine that have lower GLUT2 than pancreas are also relatively damaged by STZ. Due to its alkylating properties, STZ affects DNA. As a result, DNA damage leads to ROS formation and acute necrosis of pancreatic *β*-cells [36, 37]. The severity of nephropathy in STZ-treated animals depends on the genetic background and STZ dose [38, 39]. Generally, STZ is used to induce DN in mice and Sprague-Dawley, Wistar Kyoto, and spontaneously hypertensive (SHR) rats [40]. Currently, STZ is frequently used to induce diabetes in rats and mice [41] because their pancreatic *β*-cells are more sensitive to the cytotoxic effects of STZ compared to rabbits [41]. STZ competes with glucose to enter the *β*-cells. For this reason, animals should be fasted. Since rats and mice are nocturnal feeders, fasting should be started in the morning of blood sampling. Overnight fasting is more prolonged (approximately 24 h) which can activate several physiological mechanisms that may cause misreading of blood glucose, as a result of significant distress (AMDCC; http://www.amdcc.org). In order to reduce the toxic effects of STZ on other organs like the kidneys, the administrations of smaller doses over five consecutive days are suggested in mice [41, 42].

### 1.9. STZ-Induced DN in Mice

In mice, two protocols are used for DN induction. In one of these protocols, multiple low doses of STZ (40-50 mg/kg) are intraperitoneally injected for 5 days. In this protocol, STZ injection partially damages the pancreatic islets, thus triggering an inflammatory response which elicits a further loss of *β*-cell function and ultimately results in insulin deficiency and hyperglycemia [43, 44]. Although, the multiple injections of low doses of STZ have a less toxic effect than a single high dose, however, many investigators still prefer administration of a single high dose of STZ for diabetes induction in animals as this protocol produces a diabetic animal model that more closely resembles T1DM in terms of pathogenesis and morphological changes [44]. A single injection of STZ at a high dose (200 mg/kg) is directly toxic to pancreatic *β*-cells and causes diabetes rapidly. As compared to a low dose, using a high dose of STZ produces a greater cytotoxicity to pancreatic *β*-cells and collateral tissue, leading to a higher rate of incidence and severity of diabetes. Using low-dose STZ in mice has shown to induce hyperglycemia within 2 weeks and albuminuria within 5 weeks. In mice, unlike humans, the degree of hyperglycemia is not a critical determinant of DN development. But, in some studies, the level of nephropathy seems to correlate with the severity of hyperglycemia [45].

STZ-induced diabetes has been induced in DBA/2 and C57BL/6 mice [46, 47]. DBA/2 mice develop albuminuria within the 5 weeks after the diabetes induction, while the C57BL/6 mice exhibit albuminuria within the 25 weeks post injection of STZ [48]. Nevertheless, the mortality rate in diabetic DBA/2 mice is markedly higher than that in C57BL/6 mice. Although diabetic C57BL/6 mice can live for more than 45 weeks after the hyperglycemia development, the DBA/2 mice mortality rate increased 25 weeks after diabetes induction [49, 50].

### 1.10. STZ-Induced DN in Rats

A single dose of STZ (40 to 70 mg/kg i.p. or i.v.) administration in rats results in hyperglycemia within 72 hours. Nephropathy was noted in rats within 3–8 weeks after the administration of STZ [51, 52]. Male Sprague-Dawley or Wistar rats were frequently used in such experiments. There are debates over the appropriate pH, temperature, vehicle, and time table that should be considered when using STZ to induce diabetes [53]. There are protocols explaining the preparation method as well as dose and route of STZ administration [54]. However, several studies reported that STZ is unstable at neutral pH and should be dissolved in solutions of low pH before use; it has been indicated that STZ solutions of neutral pH are as stable as those of pH 4.5 [53]. Also, STZ solution is relatively stable at neutral pH at 37°C for at least one hour [35] and at pH 6.7–7.8 on ice for 30 min [53]. Anyway, STZ solution in citrate buffer (pH 4.5) is the most suitable preparation for use [35, 55, 56]. Female rats are less sensitive to STZ compared with male rats [53]. The lethal dose (LD50) of STZ is about 130 mg/kg. Since a single dose of 25 and >35 mg/kg in rats has no important effect and considering the following administration of 35 mg/kg, 25% of rats recovered from the diabetic state; the best dose of STZ for induction of stable diabetes is 55–65 mg/kg [54, 55, 57].

STZ is usually administrated through intravenous or intraperitoneal routes, and in some studies, it was given intramuscularly, subcutaneously, or even via intracardiac injections. However, intravenous administrations produce more stable diabetic models [53].

Disadvantages limiting of the use of STZ for induction of DN are the tissue toxicity seen at high doses and systemic administration. STZ has a nonspecific cytotoxic effect in the kidney. In particular, it negatively affects tubular cells causing acute kidney damage in mice and rats [58, 59]. STZ has carcinogenic properties, and precautions should be taken during its preparation [9].

### 1.11. Alloxan-Induced DN

Alloxan and dialuric acid (the reduced product of alloxan) generate superoxide radicals during redox cycle reactions. Then, Fenton reaction ensues with the formation of hydrogen peroxide and hydroxyl radicals which ultimately damage the pancreatic tissues in animals such as rats, mice, rabbits, and dogs [60]. Alloxan is administered intravenously, intraperitoneally, or subcutaneously [34]. For alloxan, the route of administration determines the dose. For example, intravenous injection of alloxan at the dose of 65 mg/kg is the most frequently used dose of alloxan for diabetes induction in rats. If alloxan is given intraperitoneally or subcutaneously, 2-3 times higher doses should be considered compared to the intravenous dose (i.e., >150 mg/kg. b.w) [61]. As mentioned above, alloxan has a similar structure with glucose; therefore, fasted animals are more susceptible to alloxan. Because of its lower efficacy, alloxan is less frequently used for diabetes induction, compared to STZ [62]. Diabetic nephropathy develops within 3-4 weeks after injection of alloxan [63].

### 1.12. Genetic-Chemical Model of Type 1 Diabetic Nephropathy (T1DN)

Since the renin-angiotensin-aldosterone system (RAAS) has an important role in development of human DN, several studies have used a transgenic rodents with overactivated RAAS to induce hypertension and accelerated DN [64]. Therefore, new mouse models are developed that rapidly develops the pathological features of advanced DN.

### 1.13. HD-STZ Mice: Administration of Low-Dose Streptozotocin to TTRhRen Mice

The TTRhRen mice are the transgenic animals, which the human renin cDNA transfected into their genome, and they develop renin-dependent hypertension. For diabetes induction in TTRhRen mice, STZ is administrated at the dose of 40-50 mg/kg (HD-STZ) [65]. Advanced glomerular scarring, renal hypertrophy, and tubulointerstitial fibrosis are displayed in HD mice [16, 66].

### 1.14. HD-OVE Mice: (TTRhRen) Mice×OVE26 Diabetic Mice

Diabetes induced in TTRhRen mice by intercrossing with OVE26 diabetic mice (HD-OVE mice) results in a significant nephropathy and decline in renal function. Nephropathy in both HD-OVE and HD-STZ mice is more severe than that observed in STZ-induced diabetic and OVE26 mice.

#### 1.14.1. TGR

The transgenic (mREN-2)27 rats (TGR) are the Sprague-Dawley rats where the *Ren-2* mouse renin gene has been transfected into their genome. TGR rats display an amplified tissue RAS and severe hypertension [65]. TGR rats which exhibit increased tissue renin expression are administered STZ at 55 mg/kg doses. Administration of TGR rats with STZ (55 mg/kg) provides a unique model to investigate whether an amplified tissue RAS has a role in the microvascular complications in diabetic rats [67]. Albuminuria increased significantly in 12 weeks after of STZ injection [65].

### 1.15. Surgical Models of Type 1 Diabetic Nephropathy (T1DN)

Type 1 or type 2 diabetes can be also induced by complete or partial pancreatectomy in animals, respectively. Currently, this method is rarely used in the diabetes research. Few researchers have employed this method to develop diabetes in rats, pigs, dogs, and primates. For induction of type 1 diabetes, pancreas should be completely removed [68]. A high risk of infection, as well as need for adequate antibiotic and analgesia administration after operation, technical expertise and appropriate surgical conditions, malabsorption, and loss of pancreatic response to hypoglycemia, are challenges limiting for the use of this approach [69–71].

### 1.16. Surgical+Chemical or Diet Interventions for Induction of T1DN

It was shown that hypertension promotes the development of DN; therefore, researchers attempted to induce hypertension in animal models [67]. Uninephrectomy, which can be readily applied in most animal models of DN, is an alternative method of applying hemodynamic stress. Unilateral nephrectomy in diabetic rodents accelerates the progress of DN and induces albuminuria, mononuclear inflammatory cell infiltration, and fibrosis [72, 73]. However, results from these studies showed that uninephrectomy-induced hemodynamic abnormalities may not mimic the pathophysiology of human DN [74]. Urinary expression of nephrin and podocin and GBM thickening significantly increased at week 4 after diabetes onset [75].

A high-protein diet was used to increase glomerular pressure and injury in diabetic rats in 1980 studies, but now, it is not used [76, 77].

### 1.17. Type 2 Models of Diabetic Nephropathy (T2DN)

Unlike the T1D in T2D, insulin levels can be elevated, normal, or low. Moreover, T2D is associated with the risk factors of cardiovascular disease (CVD1) including hypertension, dyslipidemia, obesity, insulin resistance, and glucose intolerance. These conditions are components of the metabolic syndrome, also known as syndrome X [78].

### 1.18. Genetic Models of Type 2 Diabetic Nephropathy (T2DN)

#### 1.18.1. Otsuka Long-Evans Tokushima Fatty Rats (OLETF)

OLETF rats are an animal model of type 2 or non-insulin-dependent diabetes mellitus (NIDDM) that is characterized by deficiency in CCK-A receptors, hyperphagia, increase in meal size, mild obesity at about 6 weeks of age, late-onset insulin resistance at around 12 weeks, NIDDM at around 30 weeks, and DN induced after 30 weeks [79].

The Zucker diabetic fatty rats (ZDF) were established by cross breeding between obese Zucker fa/fa rats and Wistar Kyoto rats, which possess leptin-receptor mutation and insulin resistance, respectively. ZDF harbors mutations of leptin receptors leading to obesity, insulin resistance, hyperglycemia, dyslipidemia, hypertension, and nephropathy. These rats become diabetic due to the loss of the *β*-cell mass. The failure of *β*-cell mass expansion may be due to gluco/lipotoxicity [80]. This model, which exhibits impaired insulin secretion and glucose tolerance, develops overt diabetes as early as 8 weeks of age, and the level of albuminuria in male ZDF rats is slightly increased at 6 weeks of age. ZDF rats develop DN characterized by heavy proteinuria and glomerulosclerosis, as early as 16 weeks of age, and after 10 weeks of age, ZDF rats developed marked albuminuria [81].

The obese ZSF1 rats are hybrid animals that are generated by crossing ZDF female rats (ZDF-/fa) with lean male rats with spontaneously hypertensive heart failure (SHHF). The ZSF1 rats exhibit signs of hyperlipidemia, hypertension, and diabetes (i.e., metabolic syndrome) at 8 weeks of age and develop nephropathy at 32 weeks of age which is characterized by massive proteinuria, severe tubulointerstitial and vascular damage, glomerulosclerosis, and reduced GFR [82]. ZSF1 rats develop more severe hypertension and renal disease as compared to the paternal ZDF diabetic rats, while do not develop hydronephrosis that may complicate evaluation of renal function and structure [82].

The Goto Kakizaki (GK) rats are considered a model of nonobese, normotensive, spontaneous, and moderate NIDDM type II [83]. The GK rats were introduced by Goto and associates at Tohoku University in Sendai by selecting breeder pairs with the highest blood glucose levels chosen based on a glucose tolerance test (OGTT) from a stock of Wistar rats over many generations [84]. GK rats exhibit impaired glucose metabolism and insulin secretion and insulin resistance. GK rats develop mild hyperglycemia and hyperinsulinemia at 3-4 weeks of age [85]. While GK rats develop glomerular hypertrophy and glomerular and tubular basement membrane thickness, they are relatively resistant to DN development and do not develop progressive proteinuria, glomerulosclerosis, or interstitial fibrosis. Recently, a substrain of GK rats was established from a cross breeding between GK and Fawn Hooded-hypertensive (FHH) rats, which develops overt diabetes and progressive proteinuria [83] by 12-13 weeks of age [22].

Ob/ob mice are leptin deficient but possess intact leptin signaling pathways. Mutation exists in C57BL/6, C57BLKS/J, FVB/N, and DBA2 strains [86–88]. Ob/ob mutation in C57BLKS/J causes *β*-cell atrophy and severe hyperglycemia. Ob/ob C57BL/6 mice develop obesity and mild hyperglycemia but do not develop the renal lesions characteristic of human diabetes [48]. Ob/ob BTBR mice exhibit sustained hyperglycemia by 6-10 weeks of age [89]. Unlike ob/ob C57BL/6J, ob/ob BTBR mice develop some pathological features of human DN [90]. BTBR ob/ob mice not only do not develop hypertension but also are rather slightly hypotensive in contrast to the majority of patients with DN. However, destruction of podocytes and onset of proteinuria are detectable by 8 weeks of age [90]. Generally, diffuse and nodular lipohyaline changes in the glomerulus are the characteristic of renal pathology in the ob/ob mice. The advantages of BTBR ob/ob mice as a model of DN are the relatively short time required for DN development and developing some pathological features of human DN. Disadvantages of this model are difficulties in breeding and high mortality rates beyond 24 weeks of age, which limit their use in evaluation of advanced nephropathy [91].

In db/db mice model, spontaneous or genetically engineered mutations in the leptin receptor gene leading to a defective leptin-signaling pathway in the hypothalamus develop an obese phenotype. Similar to the ZDF rats, db/db mice develop hyperphagia, early-onset obesity, hyperglycemia, dyslipidemia, insulin resistance, hypertension, and nephropathy, and they are infertile and deficient in growth hormone. The first-time db mutation was recognized in the C57BLKS/J strain, as well as in C57BL/6, C57BLKS, DBA, FVB, and CBA strains. Hyperglycemia is less severe in db/db and ob/ob mice with C57BL/6J background, whereas C57BLKS background develops fulminant diabetes beyond 24 weeks of age [92, 93]. Like ob/ob mice, db/db mice are insulin-resistant and develop obesity and diabetes while they are leptin-resistant in contrast to ob/ob mice. Since the db/db mice have a mutated leptin receptor, their leptin levels are markedly elevated; nevertheless, these mice suffer from morbid obesity. The db/db mouse develops significant DN compared to ob/ob mice [94]. This may be due to their different background or the lack of circulating leptin in the ob/ob mice, because leptin was demonstrated to directly stimulate matrix production. DN in these models is manifested by mesangial matrix expansion including increases in the extracellular matrix proteins such as fibronectin, type IV collagen, and laminin by 25 weeks of age [95].

Tubular atrophy, dilatation, apoptosis, and early interstitial fibrosis reflected by an increase in interstitial volume are evident at about ≥6 month post diabetes induction [95–97]. Generally, db/db mice do not develop nodular mesangial sclerosis or progressive renal failure. Hence, these mice are a good model of diabetes and early stage of DN in human but fail to display advanced features of DN. Moreover, this model fails to display advanced features of DN and requires longer periods for DN development.

The New Zealand obese (NZO) mous strain has a polygenetically inherited form of diabetes. The NZO mice develop obesity, hyperinsulinemia, hyperglycemia, glucose intolerance, and insulin resistance and thus are a useful model for studying of insulin-resistant diabetes [98]. NZO mice display progressive renal pathological features as evidenced by GBM thickness and diffuse and nodular expansion of the mesangial matrix at 24 weeks of age [99].

NONcNZO10/LtJ mouse is an inbred recombinant congenic strain generated by cross breeding between the New Zealand Obese mouse (NZO/HlLt) and the non-obese-non-diabetic (NON/LtJ) mice, which provides a model of polygenic type 2 DM male NONcNZO10/LtJ mice that are fed with a diet containing 10-11% fat (percentage of total weight), display adulthood obesity (i.e., after 13 weeks), hyperglycemia, insulin resistance, and dyslipidemia. Notably, after approximately 8 months of age, these mice also develop intensive albuminuria and, after 1 year, develop urine ACRs greater than 1000 *μ*g/mg [100, 101].

Some of the glomerular histopathologic features in this model (e.g., intraglomerular capillary thrombi and lipid deposition and periarteriolar lymphoid infiltrates), are abnormal and do not resemble human DN [98].

**MKR** mice express dominant-negative mutant insulin-like growth factor 1 receptor (IGF-1R) in skeletal muscle, are lean yet hyperlipidemic, hyperinsulinemic, and hyperglycemic, and develop insulin resistance in skeletal muscle as well as liver and fat tissues. The MKR mice are a novel murine model of DN. This model exhibits increased GFR at 10 weeks of age and a significant albuminuria at 30 weeks of age [102].

Tsumura Suzuki Obese Diabetes mice (TSOD) are obese mice with urinary glucose established by selective breeding of obese ddy strain male mice with spontaneous DM, in 1992 [103]. These mice show hyperphagia and polydipsia when they are 2 months old; also, hyperglycemia, hyperinsulinemia, hyperlipidemia, and obesity are evident as they become 12 months old [104]. Pancreatic histopathological examination of the TSOD mice showed severe hypertrophy with the proliferation of *β*-cells and complete or partial degranulation of *β*-cells [104]. In the kidney, the expansion of mesangial area and thickening of the GBM were observed in TSOD mice of >18 months old [104].

KK (Kuo Kondo) mice were bred for their large body size. This strain gradually becomes obese during adulthood and shows insulin resistance and compensatory hyperinsulinemia as a result islet cell hyperplasia [23]. In these mice, DN pathological features include mild peripheral GBM thickening, mesangial enlargement, and hypercellularity. Proteinuria and microalbuminuria were also observed in KK mice [23]. KK mice produce transgenic variants; however, this model needs prolonged periods to develop diabetes and does not develop advanced DN. It was reported that changing in diet or environmental conditions would produce overt diabetes in these mice [105].

Nishimura established genetically obese KK mice by introducing the yellow obese A^y^ gene into the KK mouse strain. The KKA^y^ mice are a cross between obese yellow male A^y^ mice and black KK female mice and carry a heterozygous mutation of the agouti gene. This strain presents severe obesity, hyperlipidemia, insulin resistance, and insulin deficiency at the age of about 8-12 weeks [106, 107]. As early as 4 weeks of age, diabetic mice show diffuse glomerulosclerosis, nodular changes, and significant elevation of albuminuria, which becomes progressively greater during diabetes progress [108].

The KKA^y^/Ta mice are obese-diabetic mice that exhibit hyperinsulinemia, hyperglycemia, and dyslipidemia. The KKA^y^/Ta mice produced by transfection of the yellow obese gene (Ay) into KK/Ta mice, a model of type 2 DN. The KKA^y^/Ta mice are a suitable model for the studying of type 2 DN because the renal injury in KKA^y^/Ta mice closely resembles the human DN. The urinary albumin/creatinine ratio (ACR) in these mice doubles from week 8 to 12 [109].

KKA^y^/Ta mice show extracellular mesangial matrix (ECM) and proliferative glomerular nephritis with expansion of glomerular at approximately 16 weeks of age [109, 110].

### 1.19. Chemical Models of Type 2 Diabetic Nephropathy (T2DN)

#### 1.19.1. Induction of DN Using STZ and Nicotinamide

This model uses consecutive administration of STZ and nicotinamide (NA) which is a niacin (B3) derivate that protects *β*-cells against toxic effects of STZ through several mechanisms including scavenging of free oxygen radicals, restoration of NAD^+^, induction of NO production, inhibition of poly ADP-ribose polymerase (PARP) and cytokine-induced MHC class II expression, regeneration of *β*-cell, and inhibition of islet cell apoptosis. This model establishes stable and moderate hyperglycemia (nonfasting) associated with the reduction of *β*-cells (40-60%) and insulin secretion but does not induce insulin resistance and glucose intolerance [34]. To induce this model, different doses of STZ (45 to 65 mg/kg) and NA (60 to 290 mg/k) were used. Importantly, NA must be injected 15 min before the administration of STZ [74]. Given that STZ exerts direct renal toxicity, it is difficult to distinguish if renal dysfunction occurs due to direct STZ toxicity or as a diabetes-induced complication. The former hypothesis can be supported by the findings suggesting that NA could prevent renal dysfunction, thereby shedding doubt on the suitability of this model for studying DN [111]. The onset of proteinuria is detectable by 8 weeks of age [112, 113].

#### 1.19.2. Neonatal STZ Diabetic Rat

A single high dose of STZ in adult rat caused induction of type 1 diabetes; however, injection of single dose of STZ at the dose range of 80-100 mg/kg to neonatal rats (2-5 day old) leads to the induction of type 2 diabetes in adult ages. Albuminuria was noted in rats by 12-20 weeks of age [114, 115].

### 1.20. Induction of Type 2 Diabetic Nephropathy (T2DN) by Modification of the Diet

#### 1.20.1. Fructose-Fed Rats

As an insulin-resistant and hypertensive model, these rats display the characteristics of metabolic syndrome. Intracellular glucose is reduced by aldose reductase to sorbitol in the polyol pathway, and then, sorbitol is oxidized to produce fructose by sorbitol dehydrogenase. In STZ-induced diabetic rats, activation of the polyol pathway increases fructose levels in the kidney and other organs. The development of diabetic complications could be triggered by accumulation of fructose, and these effects could be ameliorated by sorbitol dehydrogenase inhibition and blockade of fructose production. Fructose is administrated in two forms, in drinking water (10% or 20% *W*/*V*) or as 60% fructose diet [116–118]. Feeding with high levels of fructose directly results in hypertension, probably through inducing inflammation. Experiments showed that plasma levels of insulin and the hydrogen peroxide (as a free radical) were increased in rats fed with high fructose. Fructose-induced metabolic syndrome, either through administration of fructose in drinking water or diet, is associated with renal disorders characterized by arteriolopathy, renal hypertrophy, and glomerular hypertension at the 6-8 weeks of treatment [119].

### 1.21. High-Fat Diet-Induced Type 2 Diabetic Mice

Several reports have been indicated that the risk of chronic kidney disease is relatively high in the patients with metabolic syndrome disease [120, 121]. Feeding the animals with high-fat diet induced several systemic alterations similar to human metabolic syndrome and exhibited several types of kidney injuries [122]. Feeding HFD (60% fat) for 12 week to C57BL/6 mice has been reported to induce metabolic syndrome (indicated by obesity, hyperinsulinemia, hyperglycemia, hypertriglyceridemia, and hypertension) and subsequent kidney injuries including albuminuria, increase in glomerular tuft area, and increased accumulation of type IV collagen in glomeruli, mesangial expansion, and impaired sodium handling [123]. Overall, the genetic background can determine predisposition to the development of metabolic syndrome following HFD feeding. For instance, some strains such as C3H/He, A/J, and 129Sv mice are resistant to obesity and diabetes development while the C57BL/6 mice are considered susceptible to obesity and insulin resistance. Furthermore, the source and composition of fat is also influence the renal injury development [123]. Therefore, these factors must be taken to account before using the HFD as a model of DN.

### 1.22. Induction of (T2DN) by Administration of Chemicals+Modification of the Diet

#### 1.22.1. High-Fat Diet+Low Dose of STZ

Administration of a high-fat diet is not commonly used for DN induction since the animals rarely develop overt hyperglycemia due to the compensatory hyperinsulinemia; however, this protocol is useful for investigating the underlying mechanisms of insulin resistance [124]. According to Buettner et al., in animals, using the semi-purified high-fat diets containing more than 40% of energy based on animal fats, supplemented with a low amount of n-3 fatty acids and a low amount of plant oils rich in n-6 and n-9 fatty acids, is the best method to induce obesity in animals [125].

High-fat diet contains 49.5% fat (g/100 g of total dry diet) corresponding to 72% of the total energy content. To induce insulin resistance and decline plasma concentration of insulin as well as human diabetes, a low dose of STZ (35 mg/kg) that causes the initial *β*-cell dysfunction is given [126–128]. Following 2-4 weeks of dietary intervention, rats are injected with a low dose of STZ. Approximately 4 weeks after STZ injection, renal injury including increasing albuminuria, kidney index, and pathological changes can be observed [129].

### 1.23. Induction of (T2DN) by Administration of Chemicals, Surgical Interventions, and Modification of the Diet

#### 1.23.1. Low Dose STZ+High-Fat Diet+Nephrectomy

Sugano et al. reported that performing of nephrectomy following the low-dose STZ injection results in mild glucose intolerance in rats. Furthermore, when these rats fed with high-fat diet, they develop a rat model similar to DN seen in patients with type 2 DM. Microalbuminuria occurs at 15 weeks after STZ injection, and HFD feeding and overt proteinuria, mesangial matrix proliferation, and interstitial edema develop at 20 weeks [130].

The dose of STZ (25-40 mg/kg), level of fat (40-58% calories as fat) in diet, and percentage of nephrectomy are variable [130–132].

#### 1.23.2. Monogenic Manipulations of eNOS Deficiency

Vascular eNOS activity is altered in diabetes. It was reported that in an early diabetic state, NO production increases; however, with prolonged diabetes, renal eNOS production decreases (10). eNOS knockout (eNOS−/−) mice are used to determine whether deficiency in eNOS results in a mouse model of DN. Various types of diabetes models deficient in eNOS activity were generated. Type 1 model of DN include STZ-induced diabetic B6-*eNOS*-/- mice (low dose and high dose), B6-*eNOS*−/−*Ins2Akita*/+ mice [133, 134]. Compared to *eNOS*−/− mice, diabetic *eNOS*−/− mice develop a 10-fold increase in diabetic albuminuria following administration of low-dose STZ and a 40-fold increase following administration of high-dose STZ. DN development in *eNOS*−/−*Ins2Akita*/+ diabetic mice depends on the strain. B6-*eNOS*−/−*Ins2Akita*/+ mice die soon after weaning and therefore do not live long enough to develop DN. However, the first generation (F1) between B6 and 129SvEv survive as long as diabetic B6- *eNOS*+/+*Ins2Akita*/+ mice and develop DN [135].

Type 2 model of DN include C57BLKS (BKS)-db/db (*eNOS*−/− mice on the BKS background). Hyperglycemia is apparent at 6-8 weeks of age, and nephropathic changes appear by 16-20 weeks. These changes include hypertension, significant albuminuria, GBM thickening, mesangial expansion, nodular glomerulosclerosis, and tubulointerstitial injury as well as decreased GFR [136].

#### 1.23.3. Bradykinin B2 Receptor Deficiency

Several studies reported an association between the RASS and onset and progression of DN. Moreover, it has been reported that angiotensin-converting enzyme (ACE) inhibitors and angiotensin receptor blockers have renal protective functions in DN [137, 138].

An increase in ACE gene expression (50% in mice) significantly decreases bradykinin levels while has minimal effects on angiotensin II levels, suggesting that bradykinin plays a more important role in renal responses in diabetes than angiotensin II. Therefore, for evolution of bradykinin role in DN, investigators targeted deletion of the bradykinin 2 receptor (B2R) in *Ins2Akita*/+ mice with a B6 background [139]. *Ins2Akita*/+ mice knockout for B2R develop a 4-fold increase in albuminuria and intense mesangial expansion and glomerulosclerosis, by 6 months of age. Other investigators reported that the nonpeptide bradykinin 2 receptor antagonists in diabetic rats and BKS-db/db mice reverse almost all of the salutary effects of ACE inhibitors on renal function and albuminuria. Activation of B2R protects the kidney against DN both in 129S6/SvEvTac mice and in low-dose STZ-induced diabetic mice with B6 background. By the end of the 6-month period, more albumin excretion occurred in the STZ diabetic mice lacking B2R compared to STZ diabetic mice with intact B2R receptors [140, 141].

## 2. Limitations

Animal models of DN are frequently used to investigate the pathogenesis of DN and the therapeutic efficacy of potential treatments of diabetes-induced kidney failure. However, so far, no model has been developed to mimic all features of human DN. This review is aimed at introducing and discussing different rodent models of DN, their features, and the time to exhibit DN symptoms, to help researchers in choosing an appropriate model for their specific experiment. However, some limitations need to be acknowledged. For instance, only rodent models of DN were reviewed but there are other models using different species of animals such as rabbits, dogs, and monkeys, which were not discussed in here.

## 3. Conclusions

In summary, animal models have certain limitations in terms of contributing to our understanding of the pathogenesis of DN because no model encompasses all of the characteristic features of human DN. Few models develop structurally advanced DN; among these, OVE26 mice, *ob/ob* mice, eNOS−/−/*db/db* mice, and OLETF rats seem to be the most robust. Different genetic backgrounds and strains show different levels of susceptibility to DN with respect to albuminuria and development of morphological lesions. On the other hand, the C57BL/6 strain is relatively resistant to DN and exhibits less nephropathy features in different models of diabetes. Overall, studies have shown that in the animal models, being susceptible to DN is mostly inherited, and the triggering event of DN development is hyperglycemia. Therefore, the type of diabetes, development method of nephropathy, duration of the study, cost of maintaining and breeding, and animals' mortality rate are important factors that might be affected by the type of DN model. Furthermore, it will be particularly helpful to elucidate if renal function aggravates in any of these DN models.

## Figures and Tables

**Table 1:** Features of renal injuries in some rat and mouse models of type 1 DN.

Model	Type1	Description	Features of renal injury	Ref.
Genetically	Akita mice	Mutations in the insulin gene, causes misfolding of insulin protein	☑ Present only in C57BL/6, relatively resistant to nephropathy; hyperglycemia in females is mild; GBM thickening do not develop advanced human DN (mesangiolysis or nodular mesangial sclerosis)	[28, 29]
	OVE26 mice	Overexpression and accumulation of calmodulin in pancreatic *β*-cells leading to destruction of *β*-cells and deficient production of insulin	☑ Overt DN in FVB strain, do not develop advanced human DN on other strain	[31, 33]
	NOD mice	Autoimmune insulitis caused by polygenes including specific MHC class II alleles and many non-MHC loci	☑ Enlarged glomeruli and mesangial sclerosis	[20, 21]
	BB rat	Insulin deficiency due to autoimmune destruction of *β*-cells in Wistar rat	☑ Enhanced GFR, thickening of GBM, NO significant albuminuria, or mesangial expansion	[23, 24]

Chemical	STZ	In rats, i.v or i.p injection of STZ (40 to 70 mg/kg), and, in mice, i.v or i.p injection of STZ (100-200 mg/kg) leads to diabetes induction	☑ DN seems to correlate with the severity of hyperglycemia, albuminuria, and mesangial expansion	[43–47, 49, 51, 52]
	Alloxan	In rats, i.v or i.p injection of alloxan (40 to 200 mg/kg), and in mice, i.v or i.p injection of alloxan (50-200 mg/kg) leads to diabetes induction	☑ Enhanced GFR, glomerular hypertrophy, albuminuria, and mesangial expansion	[60–62]

Genetical+chemical	*TGR*	The transgenic (mREN-2)27 rat (TGR) with the mouse Ren-2 gene transfected into the genome of a Sprague-Dawley rat displays an amplified tissue RAS	☑ GBM thickening, mesangial hypertrophy, and occluded glomerular capillary lumens	[67]
	HD-OVE	TTRhRen mice (have the human renin cDNA) +40-50 mg/kg of STZ	☑ Renal hypertrophy and advanced glomerular scarring and tubulointerstitial fibrosis more severe than those observed in STZ-induced diabetic mice	[4, 65, 66]
	HD-STZ	TTRhRen mice×OVE26 mice	☑ Renal hypertrophy and advanced glomerular scarring and tubulointerstitial fibrosis more severe than those observed in OVE26 mice	[4, 65, 66]

Surgical+chemical	Uninephrectomized rat model of STZ-induced DN	STZ 65 mg/dl is injected before nephrectomy or after that (3 weeks after of surgery)	☑ GBM thickening, albuminuria, mononuclear inflammatory cell infiltration, and fibrosis	[72, 73, 142]

**Table 2:** Features of renal injuries in some rat and mouse models of type 2 DN.

Model	Type 2	Description	Features of renal injury	Ref.
Genetically	Goto-Kakizaki (GK)	GK rat has a decreased *β*-cell mass and insulin resistance	☑ Glomerular hypertrophy and GBM thickening, segmental glomerulosclerosis, and tubulointerstitial fibrosis, relatively resistant to the development DN	[83–85]
	(Otsuka Long-Evans Tokushima Fatty rat) OLETF	Deficiency of CCK-A receptor in the pancreas	☑ Hypertension, glomerular hypertrophy, GBM thickening, extracellular matrix expansion, nodular lesions, diffuse glomerulosclerosis, and severe tubulointerstitial fibrosis and macroalbuminuria, glomerulosclerosis in these models appears in older rats, mild obesity, late-onset hyperglycemia	[79]
	Zucker diabetic fatty rats (ZDF)	Obese Zucker fa/fa rats×Wistar Kyoto rats, possess leptin-receptor mutation	☑ Heavy proteinuria and glomerulosclerosis tubulointerstitial and vascular damage	[80, 81]
	ZSF_1_	ZDF×SHHF	☑ Massive proteinuria, glomerulosclerosis, severe tubulointerstitial, vascular damage, and reduced GFR	
	Ob/ob mice	Ob/ob mutation (a recessive mutation in the gene coding leptin), they are developed through *β*-cell atrophy and severe hyperglycemia	☑ Destruction of podocytes, proteinuria and diffuse and nodular lipohyaline	[86–90]
	Db/db mice	Mutations in the leptin receptor (similar to the Zucker rat)	☑ Glomerular hypertrophy, mesangial matrix expansion, GBM thickening do not develop advanced human DN (mesangiolysis or widespread marked or nodular mesangial sclerosis)	[92–94]
	MKR mice	Lack insulin-like growth factor 1 receptor	☑ Increased GFR, exhibit significant albuminuria nephropathy be exacerbated by uninephrectomy or high-fat diet	[102]
	Tsumura Suzuki Obese Diabetes mouse (TSOD)	Selective breeding of obese ddy strain with spontaneous DM	☑ Thickening of the glomerular basement membrane and mesangial area expansion were observed	[103, 104]
	KK mice	Is a hyperinsulinemic model that only displays mild renal pathology	☒Needs prolonged periods to develop diabetes and does not develop advanced DN	[23, 105]
	KKA^y^ mice	KK mice×yellow obese A^y^	☑ Significant elevation of albuminuria becomes progressively greater during diabetes progress	[106, 107]
	KKA^y^/Ta mice	Produced by transfection of the yellow obese gene (A^y^) into KK/Ta mice	☑ Extracellular mesangial matrix and proliferative glomerular nephritis with expansion of glomerular	[109, 110]
	NZO (new Zealand obese mouse)	This mice has a polygenetically inherited form of diabetes	☑ Displays progressive renal pathological features as evidenced by GBM thickness and diffuse and nodular expansion of the mesangial matrix	[98]

Chemical	STZ-nicotinamide	STZ (45 to 65 mg/kg)+NA (60 to 290 mg/k) were used, NA must be injected 15 min before the administration of STZ	☑ Glomerular hypertrophy, leukocyte infiltration, and glomerulosclerosis	[34, 111, 112]
	Neonatal STZ	Injection of single dose of STZ at the dose range of 80-100 mg/kg to neonatal rats (2-5 day old) leads to induction of type 2 diabetes in adult ages	☑ Albuminuria, enhanced kidney index, and pathological changes	[114, 115]

Chemical+diet	Fat-fed STZ rat	Following 2-4 weeks of dietary intervention 60% fat diet+35 mg/kg STZ	☑ Renal injury including increasing albuminuria, kidney index, and pathological changes	[126–128]

Surgical+chemical+diet	High-fat diet in low-dose-STZ-treated, heminephrectomized	Heminephrectomy performed following injection STZ (25-40 mg/kg)+feeding these rats with high-fat chow (40-58% calories as fat)	☑ Overt proteinuria, mesangial matrix proliferation, and interstitial edema	[130–132]

Diet	Fructose-fed rats	Administrated of fructose in drinking water (10% or 20% *W*/*V*) or as 60% diet	☑ Either in drinking water or diet administration is associated with renal disorder characterized by arteriolopathy, renal hypertrophy, and glomerular hypertension	[78, 119]

## References

[1] Forbes J. M., Cooper M. E. (2013). Mechanisms of diabetic complications. *Physiological Reviews*.

[2] Yokozawa T., Nakagawa T., Wakaki K., Koizumi F. (2001). Animal model of diabetic nephropathy. *Experimental and Toxicologic Pathology*.

[3] Srinivasan K., Ramarao P. (2007). Animal model in type 2 diabetes research: an overview. *Indian Journal of Medical Research*.

[4] King A. J. (2012). The use of animal models in diabetes research. *British Journal of Pharmacology*.

[5] Betz B., Conway B. R. (2014). Recent advances in animal models of diabetic nephropathy. *Nephron Experimental Nephrology*.

[6] Kern T., Engerman R. (1990). Arrest of glomerulopathy in diabetic dogs by improved glycaemic control. *Diabetologia*.

[7] Maile L. A., Busby W. H., Gollahon K. A. (2014). Blocking ligand occupancy of the *α*V*β*3 integrin inhibits the development of nephropathy in diabetic pigs. *Endocrinology*.

[8] Freedman B. I., Bostrom M., Daeihagh P., Bowden D. W. (2007). Genetic factors in diabetic nephropathy. *Clinical Journal of the American Society of Nephrology*.

[9] Aitman T. J., Critser J. K., Cuppen E. (2008). Progress and prospects in rat genetics: a community view. *Nature Genetics*.

[10] Yagihashi S., Goto Y., Kakizaki M., Kaseda N. (1978). Thickening of glomerular basement membrane in spontaneously diabetic rats. *Diabetologia*.

[11] Wan L., Bagshaw S. M., Langenberg C., Saotome T., May C., Bellomo R. (2008). Pathophysiology of septic acute kidney injury: what do we really know?. *Critical Care Medicine*.

[12] Jones T. W., Chorpa S., Kaufman J. S., Flamenbaum W., Trump B. F. (1985). Cis-diamminedichloroplatinum (II)-induced acute renal failure in the rat: enzyme histochemical studies. *Toxicologic Pathology*.

[13] American Diabetes Association (2013). Diagnosis and classification of diabetes mellitus. *Diabetes Care*.

[14] Reiser J., Sever S. (2013). Podocyte biology and pathogenesis of kidney disease. *Annual Review of Medicine*.

[15] Fukuda H., Hidaka T., Takagi-Akiba M. (2015). Podocin is translocated to cytoplasm in puromycin aminonucleoside nephrosis rats and in poor-prognosis patients with IgA nephropathy. *Cell and Tissue Research*.

[16] Brosius F. C., Alpers C. E., Bottinger E. P. (2009). Mouse models of diabetic nephropathy. *Journal of the American Society of Nephrology*.

[17] Yang Y., Santamaria P. (2006). Lessons on autoimmune diabetes from animal models. *Clinical Science*.

[18] Lenzen S., Tiedge M., Elsner M. (2001). The LEW.1AR1/Ztm-iddm rat: a new model of spontaneous insulin-dependent diabetes mellitus. *Diabetologia*.

[19] Yoon J.-W., Jun H.-S. (2001). Cellular and molecular pathogenic mechanisms of insulin-dependent diabetes mellitus. *Annals of the New York Academy of Sciences*.

[20] Watanabe Y., Itoh Y., Yoshida F. (1991). Unique glomerular lesion with spontaneous lipid deposition in glomerular capillary lumina in the NON strain of mice. *Nephron*.

[21] Todd J. A., Wicker L. S. (2001). Genetic protection from the inflammatory disease type 1 diabetes in humans and animal models. *Immunity*.

[22] Kim C. S., Sohn E. J., Kim Y. S. (2007). Effects of KIOM-79 on hyperglycemia and diabetic nephropathy in type 2 diabetic Goto-Kakizaki rats. *Journal of Ethnopharmacology*.

[23] Velasquez M. T., Kimmel P. L., Michaelis O. E. (1990). Animal models of spontaneous diabetic kidney disease. *The FASEB Journal*.

[24] Basile D. P., Anderson M. D., Sutton T. A. (2012). Pathophysiology of acute kidney injury. *Comprehensive Physiology*.

[25] Mathews C. E., Langley S. H., Leiter E. H. (2002). New mouse model to study islet transplantation in insulin-dependent diabetes mellitus. *Transplantation*.

[26] Wang J., Takeuchi T., Tanaka S. (1999). A mutation in the insulin 2 gene induces diabetes with severe pancreatic beta-cell dysfunction in the Mody mouse. *The Journal of Clinical Investigation*.

[27] Susztak K., Raff A. C., Schiffer M., Bottinger E. P. (2006). Glucose-induced reactive oxygen species cause apoptosis of podocytes and podocyte depletion at the onset of diabetic nephropathy. *Diabetes*.

[28] Gurley S. B., Mach C. L., Stegbauer J. (2010). Influence of genetic background on albuminuria and kidney injury in Ins 2+/C96Y (Akita) mice. *American Journal of Physiology-Renal Physiology*.

[29] Haseyama T., Fujita T., Hirasawa F. (2002). Complications of IgA nephropathy in a non-insulin-dependent diabetes model, the Akita mouse. *The Tohoku Journal of Experimental Medicine*.

[30] Fujita H., Haseyama T., Kayo T. (2001). Increased expression of glutathione S-transferase in renal proximal tubules in the early stages of diabetes: a study of type-2 diabetes in the Akita mouse model. *Nephron Experimental Nephrology*.

[31] Zheng S., Noonan W. T., Metreveli N. S. (2004). Development of late-stage diabetic nephropathy in OVE26 diabetic mice. *Diabetes*.

[32] Xu J., Huang Y., Li F., Zheng S., Epstein P. N. (2010). FVB mouse genotype confers susceptibility to OVE26 diabetic albuminuria. *American Journal of Physiology-Renal Physiology*.

[33] Teiken J. M., Audettey J. L., Laturnus D. I., Zheng S., Epstein P. N., Carlson E. C. (2008). Podocyte loss in aging OVE26 diabetic mice. *The Anatomical Record*.

[34] Szkudelski T. (2001). The mechanism of alloxan and streptozotocin action in B cells of the rat pancreas. *Physiological Research*.

[35] Lenzen S. (2008). The mechanisms of alloxan- and streptozotocin-induced diabetes. *Diabetologia*.

[36] Rodrigues B. (2018). Streptozotocin-induced diabetes: induction, mechanism(s), and dose dependency. *Experimental Models of Diabetes*.

[37] Pari L., Sankaranarayanan C. (2009). Beneficial effects of thymoquinone on hepatic key enzymes in streptozotocin–nicotinamide induced diabetic rats. *Life Sciences*.

[38] Susztak K., Sharma K., Schiffer M., McCue P., Ciccone E., Böttinger E. P. (2003). Genomic strategies for diabetic nephropathy. *Journal of the American Society of Nephrology*.

[39] Tesch G. H., Allen T. J. (2007). Rodent models of streptozotocin-induced diabetic nephropathy (methods in renal research). *Nephrology*.

[40] Appelhoff R. J., Hill J. V., Findon G. (2010). Differential contribution of diabetes and the *Ren2* gene to glomerular pathology in diabetic (mREN-2)27 rats. *Laboratory Investigation*.

[41] Furman B. L. (2015). Streptozotocin-induced diabetic models in mice and rats. *Current Protocols in Pharmacology*.

[42] Like A. A., Appel M. C., Williams R. M., Rossini A. A. (1978). Streptozotocin-induced pancreatic insulitis in mice. Morphologic and physiologic studies. *Laboratory Investigation*.

[43] Kolb H. (1987). Mouse models of insulin dependent diabetes: low-dose streptozocin-induced diabetes and nonobese diabetic (NOD) mice. *Diabetes/Metabolism Reviews*.

[44] Kolb-Bachofen V., Epstein S., Kiesel U., Kolb H. (1988). Low-dose streptozocin-induced diabetes in mice: electron microscopy reveals single-cell insulitis before diabetes onset. *Diabetes*.

[45] Krolewski A. S., Laffel L. M. B., Krolewski M., Quinn M., Warram J. H. (1995). Glycosylated hemoglobin and the risk of microalbuminuria in patients with insulin-dependent diabetes mellitus. *New England Journal of Medicine*.

[46] Ma L.-J., Fogo A. B. (2003). Model of robust induction of glomerulosclerosis in mice: importance of genetic background. *Kidney International*.

[47] Zheng F., Striker G. E., Esposito C., Lupia E., Striker L. J. (1998). Strain differences rather than hyperglycemia determine the severity of glomerulosclerosis in mice. *Kidney International*.

[48] Qi Z., Fujita H., Jin J. (2005). Characterization of susceptibility of inbred mouse strains to diabetic nephropathy. *Diabetes*.

[49] Shichinohe K., Shimizu M., Ishizaki M., Asakawa M. (1990). Kidney complications in EMC virus-induced diabetes in conventional DBA/2 male mice. *Experimental Animals*.

[50] Doi K., Matsuzaki H., Tsuda T., Onodera T. (1989). Rapid development of renal lesions in diabetic DBA mice infected with the D-variant of encephalomyocarditis virus (EMC-D). *British Journal of Experimental Pathology*.

[51] Brøndum E., Nilsson H., Aalkjær C. (2005). Functional abnormalities in isolated arteries from Goto-Kakizaki and streptozotocin-treated diabetic rat models. *Hormone and Metabolic Research*.

[52] Cai Y., Chen J., Jiang J., Cao W., He L. (2010). Zhen-wu-tang, a blended traditional Chinese herbal medicine, ameliorates proteinuria and renal damage of streptozotocin-induced diabetic nephropathy in rats. *Journal of Ethnopharmacology*.

[53] Deeds M. C., Anderson J. M., Armstrong A. S. (2011). Single dose streptozotocin-induced diabetes: considerations for study design in islet transplantation models. *Laboratory Animals*.

[54] Battell M. L., Rodrigues B., Yuen V. G., McNeill J. H. (1999). Treatment and pharmacological interventions in streptozotocin diabetes. *Experimental Models of Diabetes*.

[55] Junod A., Lambert A. E., Stauffacher W., Renold A. E. (1969). Diabetogenic action of streptozotocin: relationship of dose to metabolic response. *The Journal of Clinical Investigation*.

[56] Candela S., Hermandez R. E., Gagliardino J. J. (1979). Circadian variation of the streptozotocin-diabetogenic effect in mice. *Experientia*.

[57] Islam M. S., Wilson R. D., Joost H. G., Al-Hasani H., Schürmann A. (2012). Experimentally induced rodent models of type 2 diabetes. *Animal Models in Diabetes Research. Methods in Molecular Biology (Methods and Protocols), vol 933*.

[58] Lubec B., Hermon M., Hoeger H., Lubec G. (1998). Aromatic hydroxylation in animal models of diabetes mellitus. *The FASEB Journal*.

[59] Rüster C., Wolf G. (2010). Models of diabetic nephropathy. *Drug Discovery Today: Disease Models*.

[60] Etuk E. (2010). Agriculture and biology journal of north america. *Agric Biol JN Am*.

[61] Ghasemi A., Khalifi S., Jedi S. (2014). Streptozotocin-nicotinamide-induced rat model of type 2 diabetes (review). *Acta Physiologica Hungarica*.

[62] Hu Y.-Y., Ye S.-D. (2013). Experimental models of type 2 diabetic nephropathy. *Chinese Medical Journal*.

[63] Das J., Sil P. C. (2012). Taurine ameliorates alloxan-induced diabetic renal injury, oxidative stress-related signaling pathways and apoptosis in rats. *Amino Acids*.

[64] Manrique C., Lastra G., Gardner M., Sowers J. R. (2009). The renin angiotensin aldosterone system in hypertension: roles of insulin resistance and oxidative stress. *Medical Clinics of North America*.

[65] Thibodeau J.-F., Holterman C. E., Burger D., Read N. C., Reudelhuber T. L., Kennedy C. R. J. (2014). A novel mouse model of advanced diabetic kidney disease. *PLoS One*.

[66] Touyz R. M., Mercure C., He Y. (2005). Angiotensin II-dependent chronic hypertension and cardiac hypertrophy are unaffected by gp91phox-Containing NADPH oxidase. *Hypertension*.

[67] Kelly D. J., Wilkinson-Berka J. L., Allen T. J., Cooper M. E., Skinner S. L. (1998). A new model of diabetic nephropathy with progressive renal impairment in the transgenic (mRen-2)27 rat (TGR). *Kidney International*.

[68] Oztürk Y., Altan V. M., Yildizoğlu-Ari N. (1996). Effects of experimental diabetes and insulin on smooth muscle functions. *Pharmacological Reviews*.

[69] Rees D. A., Alcolado J. C. (2005). Animal models of diabetes mellitus. *Diabetic Medicine*.

[70] Choi S. B., Park C. H., Choi M. K., Jun D. W., Park S. (2014). Improvement of insulin resistance and insulin secretion by water extracts of *Cordyceps militaris*, *Phellinus linteus*, and *Paecilomyces tenuipes* in 90% pancreatectomized rats. *Bioscience, Biotechnology, and Biochemistry*.

[71] Masiello P. (2006). Animal models of type 2 diabetes with reduced pancreatic beta-cell mass. *The International Journal of Biochemistry & Cell Biology*.

[72] Tesch G. H., Allen T. J. (2007). Rodent models of streptozotocin-induced diabetic nephropathy. *Nephrology*.

[73] Komers R., Lindsley J. N., Oyama T. T., Anderson S. (2007). CYCLO-OXYGENASE-2 inhibition attenuates the progression of nephropathy in uninephrectomized diabetic rats. *Clinical and Experimental Pharmacology and Physiology*.

[74] Betz B., Conway B. R. (2016). An update on the use of animal models in diabetic nephropathy research. *Current Diabetes Reports*.

[75] Li D., Lu Z., Jia J., Zheng Z., Lin S. (2013). MiR-124 is related to podocytic adhesive capacity damage in STZ-induced uninephrectomized diabetic rats. *Kidney & Blood Pressure Research*.

[76] Berg S., Dunger A., Kloting I. (1996). Hyperglycemia and high protein diet induce similar functional alterations in the rat kidney. *Diabetologia*.

[77] Brenner B. M., Meyer T. W., Hostetter T. H. (1982). Dietary protein intake and the progressive nature of kidney disease: the role of hemodynamically mediated glomerular injury in the pathogenesis of progressive glomerular sclerosis in aging, renal ablation, and intrinsic renal disease. *New England Journal of Medicine*.

[78] Samadi Noshahr Z., Shahraki M. R., Ahmadvand H., Nourabadi D., Nakhaei A. (2015). Protective effects of Withania somnifera root on inflammatory markers and insulin resistance in fructose-fed rats. *Reports of Biochemistry & Molecular Biology*.

[79] Moran T. H. (2008). Unraveling the obesity of OLETF rats. *Physiology & Behavior*.

[80] Vora J. P., Zimsen S. M., Houghton D. C., Anderson S. (1996). Evolution of metabolic and renal changes in the ZDF/Drt-fa rat model of type II diabetes. *Journal of the American Society of Nephrology*.

[81] Chander P. N., Gealekman O., Brodsky S. V. (2004). Nephropathy in Zucker diabetic fat rat is associated with oxidative and nitrosative stress: prevention by chronic therapy with a peroxynitrite scavenger ebselen. *Journal of the American Society of Nephrology*.

[82] Bilan V. P., Salah E. M., Bastacky S. (2011). Diabetic nephropathy and long-term treatment effects of rosiglitazone and enalapril in obese ZSF1 rats. *The Journal of Endocrinology*.

[83] Janssen U., Riley S. G., Vassiliadou A., Floege J., Phillips A. O. (2003). Hypertension superimposed on type II diabetes in Goto Kakizaki rats induces progressive nephropathy. *Kidney International*.

[84] Shafrir E. (2007). *Animal models of diabetes: frontiers in research*.

[85] Movassat J., Saulnier C., Serradas P., Portha B. (1997). Impaired development of pancreatic beta-cell mass is a primary event during the progression to diabetes in the GK rat. *Diabetologia*.

[86] Haluzik M., Colombo C., Gavrilova O. (2004). Genetic background (C57BL/6J versus FVB/N) strongly influences the severity of diabetes and insulin resistance in ob/ob mice. *Endocrinology*.

[87] Liu Y.-L., Emilsson V., Cawthorne M. A. (1997). Leptin inhibits glycogen synthesis in the isolated soleus muscle of obese (ob/ob) mice. *FEBS Letters*.

[88] Chua S., Mei Liu S., Li Q., Yang L., Thassanapaff V., Fisher P. (2002). Differential beta cell responses to hyperglycaemia and insulin resistance in two novel congenic strains of diabetes (FVB-Lepr (db)) and obese (DBA-Lep (ob)) mice. *Diabetologia*.

[89] Baan M., Krentz K. J., Fontaine D. A., Davis D. B. (2016). Successful in vitro fertilization and generation of transgenics in Black and Tan Brachyury (BTBR) mice. *Transgenic Research*.

[90] Hudkins K. L., Pichaiwong W., Wietecha T. (2010). BTBR Ob/Ob mutant mice model progressive diabetic nephropathy. *Journal of the American Society of Nephrology*.

[91] Pichaiwong W., Hudkins K., Wietecha T., Shankland S., Alpers C. (2010). Reversibility of diabetic nephropathy and podocyte loss in the BTBR ob/ob mouse. *American Society of Nephrology Annual Meeting*.

[92] Hummel K. P., Coleman D. L., Lane P. W. (1972). The influence of genetic background on expression of mutations at the diabetes locus in the mouse. I. C57BL/KsJ and C57BL/6J strains. *Biochemical Genetics*.

[93] Gärtner K. (1978). Glomerular hyperfiltration during the onset of diabetes mellitus in two strains of diabetic mice (C57BL/6Jdb/db and C57BL/KsJdb/db). *Diabetologia*.

[94] Gao Q., Wolfgang M. J., Neschen S. (2004). Disruption of neural signal transducer and activator of transcription 3 causes obesity, diabetes, infertility, and thermal dysregulation. *Proceedings of the National Academy of Sciences of the United States of America*.

[95] Koya D., Haneda M., Nakagawa H. (2000). Amelioration of accelerated diabetic mesangial expansion by treatment with a PKC *β* inhibitor in diabetic db/db mice, a rodent model for type 2 diabetes. *The FASEB Journal*.

[96] Lee S. M., Bressler R. (1981). Prevention of diabetic nephropathy by diet control in the db/db mouse. *Diabetes*.

[97] Chow F., Ozols E., Nikolic-Paterson D. J., Atkins R. C., Tesch G. H. (2004). Macrophages in mouse type 2 diabetic nephropathy: correlation with diabetic state and progressive renal injury. *Kidney International*.

[98] Leiter E. H., Reifsnyder P. C. (2004). Differential levels of diabetogenic stress in two new mouse models of obesity and type 2 diabetes. *Diabetes*.

[99] Melez K. A., Harrison L. C., Gilliam J. N., Steinberg A. D. (1980). Diabetes is associated with autoimmunity in the New Zealand obese (NZO) mouse. *Diabetes*.

[100] Cho Y.-R., Kim H. J., Park S. Y. (2007). Hyperglycemia, maturity-onset obesity, and insulin resistance in NONcNZO10/LtJ males, a new mouse model of type 2 diabetes. *American Journal of Physiology-Endocrinology and Metabolism*.

[101] Reifsnyder P. C., Leiter E. H. (2002). Deconstructing and reconstructing obesity-induced diabetes (diabesity) in mice. *Diabetes*.

[102] Mallipattu S. K., Gallagher E. J., LeRoith D. (2014). Diabetic nephropathy in a nonobese mouse model of type 2 diabetes mellitus. *American Journal of Physiology-Renal Physiology*.

[103] Hirayama I., Yi Z., Izumi S. (1999). Genetic analysis of obese diabetes in the TSOD mouse. *Diabetes*.

[104] Iizuka S., Suzuki W., Tabuchi M. (2005). Diabetic complications in a new animal model (TSOD mouse) of spontaneous NIDDM with obesity. *Experimental Animals*.

[105] Reddi A. S., Camerini-Davalos R. A., Camerini-Davalos R. A., Cole H. S. (1988). Hereditary diabetes in the KK mouse: an overview. *Prediabetes. Advances in Experimental Medicine and Biology, vol 246*.

[106] Umezawa K., Kawakami M., Watanabe T. (2003). Molecular design and biological activities of protein-tyrosine phosphatase inhibitors. *Pharmacology & Therapeutics*.

[107] Kato H., Ohue M., Kato K. (2001). Mechanism of amelioration of insulin resistance by *β*_3_-adrenoceptor agonist AJ-9677 in the KK-Ay/Ta diabetic obese mouse model. *Diabetes*.

[108] Ledbetter S., Copeland E. J., Noonan D., Vogeli G., Hassell J. R. (1990). Altered steady-state mRNA levels of basement membrane proteins in diabetic mouse kidneys and thromboxane synthase inhibition. *Diabetes*.

[109] Okazaki M., Saito Y., Udaka Y. (2002). Diabetic nephropathy in KK and KK-Ay mice. *Experimental Animals*.

[110] Hagiwara S., Makita Y., Gu L. (2006). Eicosapentaenoic acid ameliorates diabetic nephropathy of type 2 diabetic KKAy/Ta mice: involvement of MCP-1 suppression and decreased ERK1/2 and p38 phosphorylation. *Nephrology Dialysis Transplantation*.

[111] Maiese K., Chong Z., Hou J., Shang Y. (2009). The vitamin nicotinamide: translating nutrition into clinical care. *Molecules*.

[112] Ahmadvand H., Tavafi M., Khosrowbeygi A. (2012). Amelioration of altered antioxidant enzymes activity and glomerulosclerosis by coenzyme Q10 in alloxan-induced diabetic rats. *Journal of Diabetes and its Complications*.

[113] Sheela N., Jose M. A., Sathyamurthy D., Kumar B. N. (2013). Effect of silymarin on streptozotocin-nicotinamide-induced type 2 diabetic nephropathy in rats. *Iranian Journal of Kidney Diseases*.

[114] Shirali S., Zahra Bathaie S., Nakhjavani M. (2013). Effect of crocin on the insulin resistance and lipid profile of streptozotocin-induced diabetic rats. *Phytotherapy Research*.

[115] Blondel O., Bailbe D., Portha B. (1989). Relation of insulin deficiency to impaired insulin action in NIDDM adult rats given streptozocin as neonates. *Diabetes*.

[116] Shahraki M. R., Noshahr Z. S., Ahmadvand H., Nakhaie A. (2016). Anti-nociceptive and anti-inflammatory effects of Withania somnifera root in fructose fed male rats. *Journal of Basic and Clinical Physiology and Pharmacology*.

[117] Shahraki M. R., Mirshekari H., Samadi Z., Shahraki A. R., Shahraki E. (2017). Effects of *Artemisia dracunculus* aqueous extract on blood sugar, serum insulin, triglyceride and liver enzymes in fructose drinking water male rats. *Zahedan Journal of Research in Medical Sciences*.

[118] Mohammad Reza S., Hamideh M., Zahra S. (2015). The nociceptive and anti-Inflammatory effects of *Artemisia dracunculus* L. aqueous extract on fructose fed male rats. *Evidence-based Complementary and Alternative Medicine*.

[119] Sanchez-Lozada L. G., Tapia E., Jimenez A. (2007). Fructose-induced metabolic syndrome is associated with glomerular hypertension and renal microvascular damage in rats. *American Journal of Physiology-Renal Physiology*.

[120] Ninomiya T., Kiyohara Y., Kubo M. (2006). Metabolic syndrome and CKD in a general Japanese population: the Hisayama Study. *American Journal of Kidney Diseases*.

[121] Kurella M., Lo J. C., Chertow G. M. (2005). Metabolic syndrome and the risk for chronic kidney disease among nondiabetic adults. *Journal of the American Society of Nephrology*.

[122] Kume S., Uzu T., Araki S. (2007). Role of altered renal lipid metabolism in the development of renal injury induced by a high-fat diet. *Journal of the American Society of Nephrology*.

[123] Deji N., Kume S., Araki S. (2009). Structural and functional changes in the kidneys of high-fat diet-induced obese mice. *American Journal of Physiology-Renal Physiology*.

[124] Srinivasan K., Viswanad B., Asrat L., Kaul C. L., Ramarao P. (2005). Combination of high-fat diet-fed and low-dose streptozotocin-treated rat: a model for type 2 diabetes and pharmacological screening. *Pharmacological Research*.

[125] Buettner R., Scholmerich J., Bollheimer L. C. (2007). High-fat diets: modeling the metabolic disorders of human obesity in rodents. *Obesity (Silver Spring)*.

[126] Zhang M., Feng L., Zhu M. M. (2014). The anti-inflammation effect of moutan cortex on advanced glycation end products-induced rat mesangial cells dysfunction and high-glucose–fat diet and streptozotocin-induced diabetic nephropathy rats. *Journal of Ethnopharmacology*.

[127] Liu Z., Li W., Li X. (2013). Antidiabetic effects of malonyl ginsenosides from Panax ginseng on type 2 diabetic rats induced by high-fat diet and streptozotocin. *Journal of Ethnopharmacology*.

[128] Zheng X., Zhang L., Wang W. W., Wu Y. Y., Zhang Q. B., Feng W. S. (2011). Anti-diabetic activity and potential mechanism of total flavonoids of *Selaginella tamariscina* (Beauv.) Spring in rats induced by high fat diet and low dose STZ. *Journal of Ethnopharmacology*.

[129] Wu D., Wen W., Qi C. L. (2012). Ameliorative effect of berberine on renal damage in rats with diabetes induced by high-fat diet and streptozotocin. *Phytomedicine*.

[130] Sugano M., Yamato H., Hayashi T. (2006). High-fat diet in low-dose-streptozotocin-treated heminephrectomized rats induces all features of human type 2 diabetic nephropathy: a new rat model of diabetic nephropathy. *Nutrition, Metabolism and Cardiovascular Diseases*.

[131] Mansor L. S., Gonzalez E. R., Cole M. A. (2013). Cardiac metabolism in a new rat model of type 2 diabetes using high-fat diet with low dose streptozotocin. *Cardiovascular Diabetology*.

[132] Skovsø S. (2014). Modeling type 2 diabetes in rats using high fat diet and streptozotocin. *Journal of Diabetes Investigation*.

[133] Parhizgar S., Hosseinian S., Soukhtanloo M. (2018). *Plantago major* protects against cisplatin-induced renal dysfunction and tissue damage in rats. *Saudi Journal of Kidney Diseases and Transplantation*.

[134] Hosseinian S., Ebrahimzadeh Bideskan A., Shafei M. N. (2018). Nigella sativaextract is a potent therapeutic agent for renal inflammation, apoptosis, and oxidative stress in a rat model of unilateral ureteral obstruction. *Phytotherapy Research*.

[135] Zhao H. J., Wang S., Cheng H. (2006). Endothelial nitric oxide synthase deficiency produces accelerated nephropathy in diabetic mice. *Journal of the American Society of Nephrology*.

[136] Mohan S., Reddick R. L., Musi N. (2008). Diabetic eNOS knockout mice develop distinct macro- and microvascular complications. *Laboratory Investigation*.

[137] Marre M., Bernadet P., Gallois Y. (1994). Relationships between angiotensin I converting enzyme gene polymorphism, plasma levels, and diabetic retinal and renal complications. *Diabetes*.

[138] Doria A., Warram J. H., Krolewski A. S. (1994). Genetic predisposition to diabetic nephropathy: evidence for a role of the angiotensin I–converting enzyme gene. *Diabetes*.

[139] Huang W., Gallois Y., Bouby N. (2001). Genetically increased angiotensin I-converting enzyme level and renal complications in the diabetic mouse. *Proceedings of the National Academy of Sciences*.

[140] Tan Y., Keum J.-S., Wang B., McHenry M. B., Lipsitz S. R., Jaffa A. A. (2007). Targeted deletion of B2-kinin receptors protects against the development of diabetic nephropathy. *American Journal of Physiology-Renal Physiology*.

[141] Kakoki M., Smithies O. (2009). The kallikrein–kinin system in health and in diseases of the kidney. *Kidney International*.

[142] Mauer S. M., Brown D. M., Matas A. J., Steffes M. W. (1978). Effects of pancreatic islet transplantation on the increased urinary albumin excretion rates in intact and uninephrectomized rats with diabetes mellitus. *Diabetes*.

